# Vulnerability assessment of Iran's rural-farmer households during COVID-19 pandemic

**DOI:** 10.3389/fpubh.2022.994922

**Published:** 2022-09-14

**Authors:** Mohammad Shokati Amghani, Moslem Savari, Shahla Choobchian

**Affiliations:** ^1^Department of Agricultural Extension and Education, College of Agriculture, Tarbiat Modares University (TMU), Tehran, Iran; ^2^Department of Agricultural Extension and Education, Agricultural Sciences and Natural Resources University of Khuzestan, Mollasani, Iran; ^3^Department of Agricultural Extension and Education, College of Agriculture, Tarbiat Modares University (TMU), Tehran, Iran

**Keywords:** vulnerability, COVID-19, sustainable livelihoods, rural society, wheat farmers

## Abstract

The COVID-19 pandemic caused an emergency around the world, especially in rural communities, and imposed great disasters on human societies, so it's devastating effects on mental health indicators, economy, environment, and social relations are known to everyone. But the accurate assessment of its damage to human societies can help to manage this phenomenon during and post-COVID-19 pandemic. To that end, the present study was conducted for vulnerability assessment of wheat farmers to the COVID-19 pandemic in northwest Iran. The main data collection tool in this study was a questionnaire that was designed based on the Me-bar model, but for the accurate vulnerability assessment, new parameters were added based on the theoretical research literature and the COVID-19 pandemic. The sample size was selected from 420 wheat farmers living in East Azerbaijan Province, the northwest of Iran, using the Kerjcie and Morgan's table. The results showed that for economic vulnerability, the rural poverty was the most important cause of vulnerability of the studied rural households and access to information was most important cause of social vulnerability. Also, the results showed that for psychological vulnerability, the self-efficacy was the most important cause of vulnerability. In other results, irrigation parameters of agricultural lands were the most important cause of environmental vulnerability. The study results showed that the studied farmers have experienced high levels of vulnerability and were strongly affected by economic, social, psychological, and environmental damages. Moreover, the results showed that the farmers of Shabestar and Maragheh had the highest level of vulnerability. In general, the study results can provide policymakers with new insights into the field of COVID-19 pandemic management because the vulnerability of farmers has been identified using 39 parameters.

## Introduction

The COVID-19 pandemic have been a significant disorder in the global social and ecological system and has affected billions of livelihoods (1). This is an unprecedented health crisis in the history of humans, whose social and economic effects are evident around the world (2–4). Therefore, this pandemic has affected the food system globally. The incidence of this phenomenon will have the same effects on the global food system at different times (5). This disease has affected all components of the food system, including the initial supply of the product for processing, and trade, as well as the national and international transportation system and intermediate and final demand for products. As well as, this disease has affected the input market such as the labor and capital markets (5). Simultaneously with the continuation of the COVID-19 pandemic, it is necessary to identify the problems that affect the agriculture and food sector for the supply and demand and provide solutions to reduce these effects (6, 7). In the meantime, it is very important to ensure the continuity of the food supply chain at the national and global levels to ensure food security and prevent crises, especially in countries that have had food crises (8). Therefore, considering that most of the agricultural production is done in rural areas, The impact of COVID-19 on farmers' livelihoods and food security is a key concern in rural communities (9). This acute respiratory syndrome coronavirus 2 (SARS-CoV-2) was recorded for the first time in Wuhan in the People's Republic of China (10, 11) and reported by WHO on Dec. 31, 2019 (12). With the spread of news about contagious diseases, many countries attempted to close their borders and took restrictive measures in traffic (13). Limiting interactions has caused negative impacts on countries' economies and seriously disrupted agricultural systems that were responsible for food production and supply (14, 15). The COVID-19 pandemic caused many threats to sustainability in the agriculture sector, among the most important secondary effects of the COVID-19 pandemic, were workforce restrictions, delays in agricultural operations, transportation, etc. (16).

**Figure 1:**
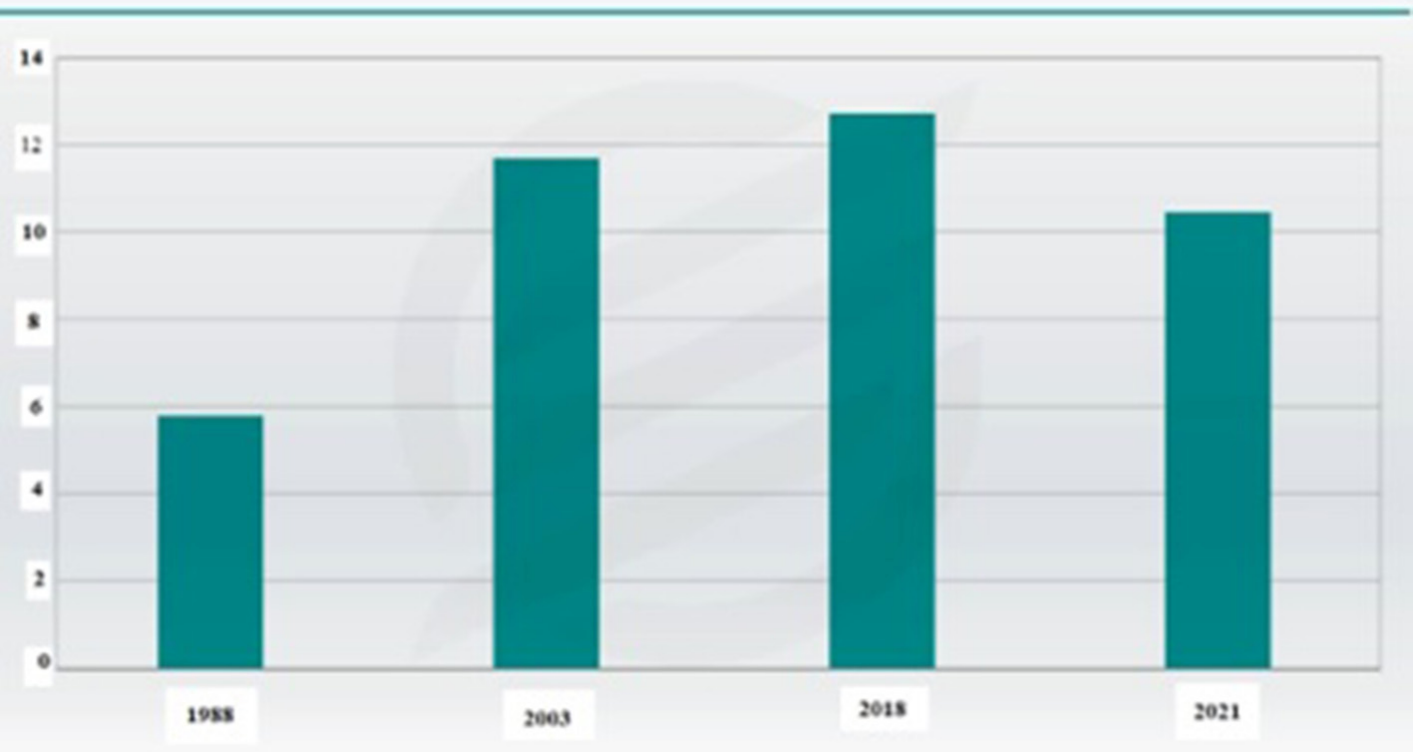
The trend of changes in the amount of annual wheat production in the past years (unit: million tons). Source: [14].

Moreover, farmers themselves have increased their level of vulnerability due to living in villages and being far from health facilities (17). The review of the import of agricultural products showed that in the second quarter of 2020, about 1.94 million tons of wheat were imported, which accounted for 28% of the total import of agricultural products in the second quarter 2020 (8). Based on the information in Figure 1 for wheat production, the data from the Iranian Statistics Center showed that the wheat production in 2021 was equal to 10 million and 440 thousand tons, which was less than in 2003 and 2018. This main reason for this could be the COVID-19 pandemic (18).

Also, according to the announcement of the Ministry of Agricultural-Jihad, the COVID-19 pandemic in 2020 caused 2 thousand 173 billion Tomans to lose to the agriculture sector of East Azerbaijan Province (19). It should be noted that due to COVID-19 pandemic problems for farmers, the negative impact on the rural economy, and negative mental and psychological impacts, the motivation of farmers for work and daily activities was reduced and as a result, production and field management had problems. What can increase the level of vulnerability of rural and agricultural households to shocks is the lack of information about their vulnerability (20). In order to clarify the issue of vulnerability and explain its economic, social, psychological, and environmental dimensions have been discussed. Therefore, the research question is What are the effects of the Covid-19 pandemic on wheat farmers in East Azarbaijan province?

## Vulnerability assessment

Vulnerability is defined as being physically or emotionally wounded (21). Vulnerability is the degree to which a system is unable to cope with a real shock or risk and falls (22). In fact, vulnerability indicates the system's failure to deal with a real shock (such as the COVID-19 pandemic) (23). Vulnerability is a function of three elements: sensitivity to stimuli, exposure to changes, and adaptive capacity (20). Sensitivity is the degree that a system is subject to or affected by stimuli (24). Sensitivity can be described as the probability of encountering different degrees of the effects of encountering stimuli (25). Encountering refers to being exposed to risks and is a set of methods of dealing with social, economic, and environmental stress (26, 27). It should be noted that in this research, the meaning of encounter is the same as exposure the conditions of the COVID-19 pandemic. Adaptive capacity is the capability of a system to apply adaptation strategies so that it can deal with social, economic, and environmental stress. Four factors are effective on the vulnerability, including physical (such as infrastructure of society such as roads, electricity, water, etc.), economic (income, and capital), social (such as education, security, and justice), and environmental factors (such as the climate of the region) (28). Of course, these factors depend on different shocks, for instance, perhaps the most important damage of the COVID-19 pandemic is the psychological effects and anxiety caused (29). In a general category, the damages caused by the COVID-19 pandemic can be classified into economic, social, environmental, and psychological factors.

### Economic vulnerability

Most of the studies on economic hazards of the COVID-19 pandemic among farmers have referred to possible loss of economic assets and processes of agricultural systems (30–35) and a number of other studies focused on the destruction of physical and social infrastructure and costs related to maintenance, waste of agricultural products, costs of agricultural inputs due to factory holidays, and reduction in farmers' income (36–38). Some of them mentioned the risks of unemployment, job loss (16, 31, 39–41), income inequality (42), increase in food costs, reduction in food diversity among households, increase in malnutrition (43), and disruption in the food supply chain (44).

### Social vulnerability

This dimension emphasizes the most vulnerable groups of society in rural areas, such as low-income people, homeless women and children, the disabled, and the elderly, as the COVID-19 pandemic has the greatest effect on them (41, 43–48). A number of studies have focused on the most important social effects of the COVID-19 pandemic on activities that require participation, social trust, and collective activities that are disrupted due to the fear of infection (29, 49) and a number of other studies pointed to the effects of COVID-19 pandemic on the stability of life and an increase in household migration due to the loss of economic power and the exhaustion of savings (17). A number of other studies have emphasized an increase in poverty and social inequality (50–52), and other researchers also pointed to the lack of access to information and knowledge to deal with COVID-19 pandemic (53, 54).

### Psychological vulnerability

Psychological vulnerability is one of the most important effects of the COVID-19 pandemic (55). Because the spread of the disease led to the closure of public, educational, recreational centers, and social quarantine, and has negatively affected the mental health of the people (55). Because the spread of the disease and the worry about the possibility of death due to viral infections increased and led to psychological vulnerability (56, 57). The studies of this section are divided into three general categories, some of them have focused on the fear of infection and the effects of quarantine and social distancing, which has caused an increase in mental diseases (56, 58), some others have focused on the effects of the death of one of the family members, job, livelihood assets, that the social vitality of the households has been lost (59, 60) and other studies have emphasized the lack of self-efficacy and the inability of farmers to cope psychologically (61).

### Environmental vulnerability

This dimension investigates the environmental damage related to the effects of the COVID-19 pandemic on soil erosion, and animal and plant loss (17). A number of studies in this section addressed the cultivation pattern, the irrigation situation in agriculture, the type of cultivation, and suitable agricultural inputs (62, 63), and a number of others emphasized the waste caused by products and the pollution caused by the waste of agricultural products in the environment (37, 38). Thus, it is clear that the contagion of an epidemic disease like Corona, in addition to health effects on rural communities, can affect economic activities, social relations, psychological aspects and the rural environment. Therefore, in order to deal with this pandemic, it is better to first conduct a comprehensive vulnerability analysis of the different aspects of the rural community in relation to the contagion of the COVID-19, and then apply the necessary strategies based on the state of vulnerability in different dimensions. Understanding the vulnerability of farmers dealing with shocks and threats cannot only help to reduce the negative impacts of the phenomena but as well as plays an essential role in the management of threatening phenomena (64). To that end, this study was conducted for vulnerability assessment of rural-farmer households in Northwest Iran dealing with the COVID-19 pandemic. The following specific objectives were followed in order to achieve this.

✓ Investigating the economic vulnerability of wheat farmers in dealing with COVID-19.
✓ Investigating the social vulnerability of wheat farmers in dealing with COVID-19.
✓ Investigating the psychological vulnerability of wheat farmers in dealing with COVID-19.
✓ Investigating the environmental vulnerability of wheat farmers in dealing with COVID-19.
✓ Investigating the total vulnerability of wheat farmers in dealing with COVID-19.

## Methodology

### Study type

This research was a quantitative study in nature, an applied study in goal, a descriptive survey in data collection, and a cross-sectional study in terms of time. It is necessary to mention that Cross-sectional studies are observational studies that analyze data from a population at a single point in time. They are often used to measure the prevalence of health outcomes, understand determinants of health, and describe features of a population (65).

### Statistical population and sampling method

The statistical population was composed of wheat farmers in the counties of Varzeghan, Maragheh, Shabestar, Sarab and Tabriz which have been exposed to Covid-19 pandemic during the last years. Kerjcie and Morgan's (66) table was used to estimate the sample size. Finally, 420 farmers engaged in wheat cultivation were selected for the study. The sampling method was random.

### Study area

**Figure 2:**
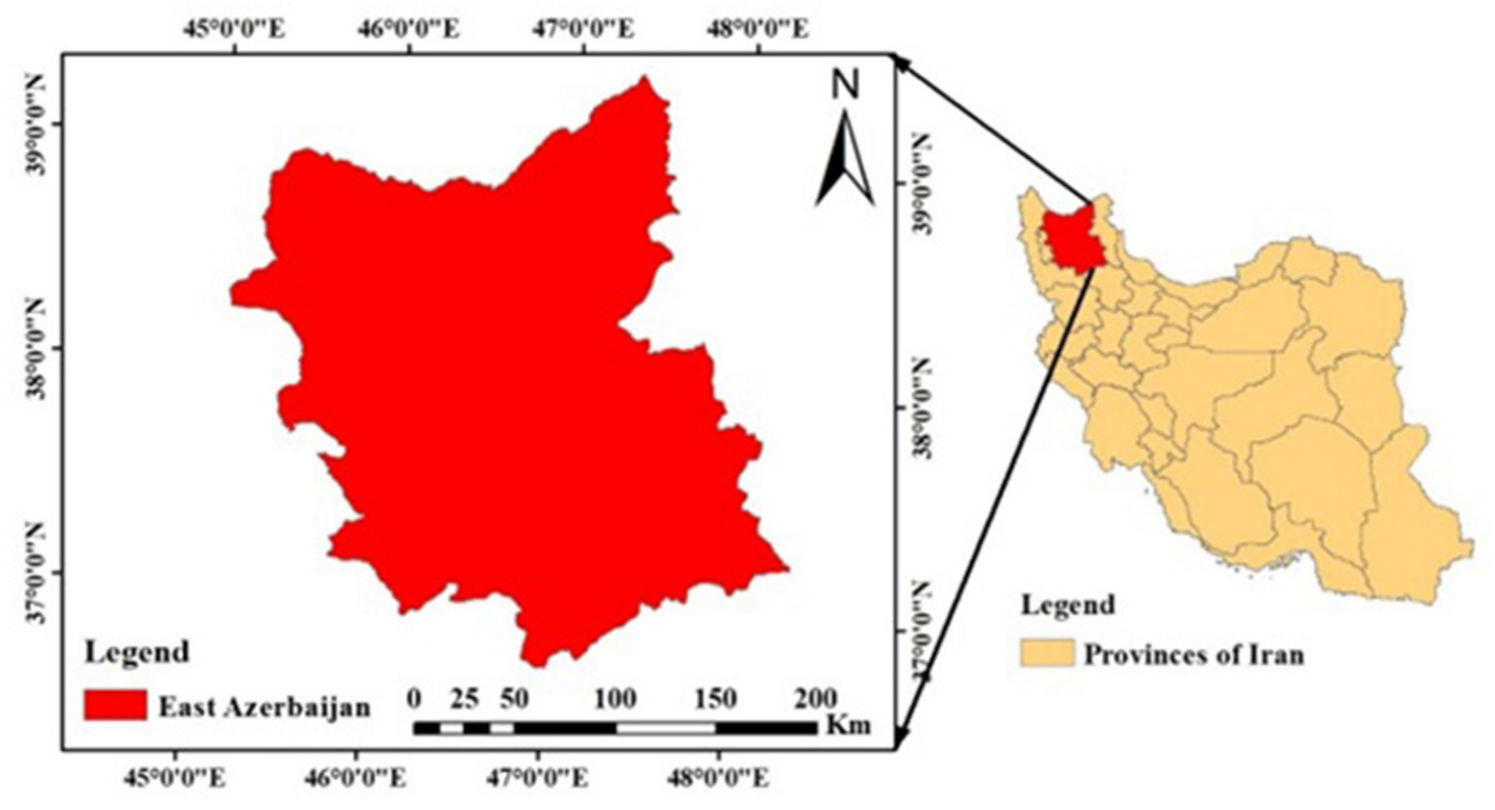
The study area.

Based on the map of East Azarbaijan Province in the format of Figure 2 East Azarbaijan Province, with an area of about 45,490.89 square kilometers and devoting 2.76% of the country's area to itself, is located in the northwestern corner of the Iranian plateau. East Azarbaijan Province has a 235 km common boundary with the Republics of Azerbaijan and Armenia. This province is bordered by Ardabil province in the west, West Azerbaijan province in the east, and Zanjan province in the south (67). According to available data, this year in East Azerbaijan, 457,000 hectares of wheat fields have been cultivated, of which 75,000 hectares are irrigated and the rest are dry. It should be noted that 70% of the wheat fields of East Azerbaijan are located in the southern part of this province and in the counties of Hashtrood, Charoymaq, Miyaneh and Maragheh, and the recent rains have been very effective in the growth of wheat in these areas. East Azerbaijan province is the sixth wheat production hub in Iran and 8% of Iran's wheat is produced in this province (19).

### Measurements

The main research instrument was a questionnaire that was mainly designed based on the formula presented by Me-Bar and Valdez (68). The questionnaire was composed of two general sections. The first section was related to the characteristics of farmers and farms. The second section included items to measure vulnerability parameters that were implemented in the scenario form. This section consisted of four parts: (i) 15 parameters for calculating economic vulnerability, (ii) 10 parameters for calculating social vulnerability, (iii) seven parameters for calculating psychological vulnerability, (iv) and seven parameters for calculating environmental vulnerability (Table 1).

**Table 1:** The main parameters and sub-parameters used in this research.

**j**	**i**			
	**1**	**2**	**3**	**4**
	**Economic**	**Social**	**Psychological**	**Environmental**
1	Rural poverty	The occurrence of crimes	Self-esteem	Agricultural production waste
2	Rural saving	Rural youth marriage	Efficacy	Cropping pattern
3	Crop selling	The stability of daily rural life	Worry and anxiety	Type of cultivation (autumn and spring)
4	Household nutrition	migration	Hope	Equipping and renovating agricultural land
5	Providing production inputs	Unity and solidarity of villagers	Risk taking	Good Agricultural Practices (GAP)
6	Crop pricing	Social dignity	Correct and decisive decision	Water channel drainage
7	Access to wage labor	access to information	Social vitality	Irrigation of agricultural lands
8	Transferring crops to the market	Dependence on others		
9	Agricultural waste	Cooperation and social participation		
10	The cost of crop producing	sympathy		
11	Supply chain of agricultural products			
12	Processing of agricultural products			
13	Non-farm incomes of rural households			
14	Crop insurance			
15	Farm income			
ki	15	10	7	7

### Validity and reliability of the instrument

To evaluate the indicators measured, the draft of the survey and questionnaire was reviewed by a panel of experts before interviewing the farmers. The panel was composed of professors in the fields of agricultural extension and education, environment, psychology, social science, and agricultural science. The questionnaire was revised based on their views until it was finally approved by the panel. The reliability of the research instrument was as well as evaluated by the coefficient of composite reliability, which had an acceptable value (up to 0.8).

### Vulnerability assessment

Me-Bar and Valdez (68) provided a formula for measuring drought vulnerability. The proposed formula is based on the subjective assessment of factors affecting vulnerability. Since the importance of parameters in the degree of vulnerability is not the same and the quality and quantity of vulnerability varies from region to region, vulnerability can be considered a function of time and place variables (69), so it seems logical to use subjective assessment methods. On the other hand, since the constituents of this formula are Pi (conditions faced by the farmer during drought) and Wi (relative importance and weight of each parameter in the amount of vulnerability), the value of Pi is determined by farmers and the amount of Wi is determined by experts. Drought vulnerability is, thus, described by people (farmers and experts) who play an important role in management planning and are somehow involved in the consequences of drought, which is a strength of the model (70). Other advantages of this model vs. the models proposed by other researchers are the explicit statement of its components and the simplicity of vulnerability calculation. Indeed, the components of the formula in some models are expressed in a very general and qualitative way, making it difficult to calculate them. In fact, using the relevant formula, vulnerability, which is a qualitative concept, can be converted into a quantitative model and the degree of vulnerability can be determined for each region and even each farmer (71). The steps for vulnerability assessment are as follows:

1. Selecting the time period and target population: In this study, the target population was wheat farmers in East Azarbaijan province in northwestern Iran who have been exposed to COVID-19 conditions from 2019 to 2021.

2. Identifying parameters affecting vulnerability: The parameters that were selected to measure vulnerability based on the literature review included economic, social, psychological, and environmental factors.

3. Calculating the value of each parameter: The parameters were calculated by items with a scale of 1–5. The farmers were asked to express their vulnerability in each of the mentioned factors. For this purpose, five options were set for each parameter as a scenario that indicated the conditions of farmers during COVID-19, in which the first option reflected the best condition and least vulnerability and the fifth option reflected the worst condition and most vulnerability.

4. Determining an appropriate scale for weighting the parameters and calculating the weight of each parameter in total vulnerability: Since the parameters are not of equal importance in explaining the degree of vulnerability and each has a specific weight, each parameter should be assigned with a weight at this stage. The weight of each parameter, denoted by Wi, indicates the relative importance of that parameter among other parameters.

To assign weights to the parameters, a questionnaire was prepared and provided to the agricultural experts and they were asked to assign a weight of 0–10 to each parameter in terms of its importance in COVID-19 vulnerability. It is important to note that Equation 1 is established for the total weight of the total vulnerability of each factor.


(1) $$\begin{array}{l} \;\sum W_{i}=C_{0}\\ C_{0}=\frac{W_{\max}\times n}{2}\\ \;C_{0}<W_{\max}\times n\\ \sum W_{i}=\frac{W_{\max}\times n}{2} \end{array}$$

In which Wmax represents the maximum weight assigned to each parameter and n represents the number of the parameters of each factor.

This condition for weight assignment prevented the evaluator to give a weight of 10 to all parameters and made them assign a suitable weight for each parameter and observe the balance in weighting the parameters. Finally, the average weight of each parameter was considered the relative importance of that parameter in the overall vulnerability. Eventually, the vulnerability of each factor was calculated using Equation 2 as follows:


(2) $$\begin{array}{r} V_{i}=\frac{1}{C_{i}}\times \overset{k_{i}}{\underset{j=1}{\sum }}\left(P_{j}\times W_{j}\right) \end{array}$$

in which V is vulnerability, Pi is the value of each parameter, Wi is the weight of each parameter, and C is the total vulnerability weight.

5. Calculating the total vulnerability: The total vulnerability (a combination of all parameters) is calculated by Equation 3.


(3) $$\begin{array}{r} V_{L}=\frac{\sum_{i=1}^{n}V_{i}\times C_{i}}{\sum_{i=1}^{n}C_{i}} \end{array}$$

6. Calculating relative vulnerability: To calculate the relative vulnerability, a place should be considered the reference and other areas should be calculated based on it by Equation 4. (Here, Tabriz was considered as the reference because it is the capital of the province and has suitable facilities vs. other areas.)


(4) $$\begin{array}{r} V_{R,L}=\frac{V_{L}}{V_{Tabriz}} \end{array}$$

It should also be noted that the opinions of 22 people experts were used to weight economic, social, psychological, and environmental vulnerability criteria, and the results are presented in the weight columns of Tables 2–5.

**Table 2:** The values and weights of farmers' economic vulnerability parameters.

**Weight (Wj)**	**Economic sub-parameters**	**Value**				
		**(Pj)**				
		**Varzeghan**	**Maragheh**	**Shabestar**	**Sarab**	**Tabriz**
		**Pi**	**Pi**	**Pi**	**Pi**	**Pi**
7/3	Rural poverty	45/3	12/3	38/4	88/3	3/3
6/56	Rural saving	52/3	85/3	24/3	49/3	8/4
5/45	Crop selling	49/3	19/3	40/3	52/3	11/4
4/63	Household nutrition	25/3	52/3	87/3	41/3	15/3
4/55	Providing production inputs	19/3	88/3	69/3	19/3	29/3
3/21	Crop pricing	12/3	48/3	58/3	44/3	4/4
4/45	Access to wage labor	2/4	59/3	11/4	10/4	22/3
4/39	Transferring crops to the market	85/3	67/3	88/3	16/3	74/3
2/25	Agricultural waste	41/3	59/3	2/3	49/3	86/3
4/55	The cost of crop producing	3/4	49/3	11/4	8/4	87/3
6/36	Supply chain of agricultural products	4/4	12/4	24/3	9/4	63/3
4/4	Processing of agricultural products	63/3	52/3	19/3	63/3	45/3
6/36	Non-farm incomes of rural households	48/3	62/4	59/4	45/4	9/4
4/2	Crop insurance	27/3	42/3	45/3	8/3	12/3
6/79	Farm income	46/3	45/4	49/3	8/4	9/4
**Total vulnerability**		34/3	50/3	48/3	49/3	26/3

P_i_: The amount of each parameter on a scale of 1 (least vulnerability) to 5 (most vulnerability) from the farmers' perspective.

W_j_: The relative importance of each parameter on a scale of 0 (the lowest weight) to 10 (the highest weight).

## Results

### Economic vulnerability

To measure economic vulnerability, the weight of total vulnerability was first calculated for all economic parameters by the following equation.


$$\begin{array}{l} \sum W_{i}{\mathrm{=C}}_{1}\\ \sum \mathrm{Wi=}(\mathrm{Wmax\times n})\mathrm{/2=}(\mathrm{10}\times \mathrm{15})\mathrm{/2=75} \end{array}$$

Table 2 shows the weight of the parameters assigned by the experts and farmers. Based on the results, it can be said that Rural poverty, agricultural income and household savings are the most important in accounting for vulnerability. In other words, these three factors are the largest source of vulnerability for wheat farmers in northwestern Iran. In addition, a glance at the economic vulnerability of wheat farmers reveals that all studied areas are at a high level of economic vulnerability as the amount of vulnerability is above the average (3 of 5) in all areas. The results also indicate that among the studied areas, Maragheh and Sarab counties are most vulnerable (Table 2).

### Social vulnerability

Table 3 presents the weights and values of the social parameters. The following formula was used to calculate the weights of the parameters in this section.


$$\begin{array}{lll} \sum \text{Wi} & = & {\text{C}}_{1}\\ \sum \text{Wi} & = & \left(\text{Wmax}\times \text{n}\right)/2=\left(10\times 10\right)/2=50 \end{array}$$

According to the results in Table 3, the parameters of access to information (6.49), cooperation and social participation (6.45) and the stability of daily rural life (6.23) have the greatest effect on the vulnerability of the studied wheat farmers. Moreover, the results showed that Shabester and Maragheh counties experienced the highest level of social vulnerability with vulnerability values of 4.22 and 4.20, respectively (Table 3).

**Table 3:** The values and weights of farmers' social vulnerability parameters.

**Weight (Wj)**	**Social sub-parameters**	**Value**				
		**(Pj)**				
		**Varzeghan**	**Maragheh**	**Shabestar**	**Sarab**	**Tabriz**
		**Pi**	**Pi**	**Pi**	**Pi**	**Pi**
2/25	The occurrence of crimes	2/98	3/21	3/42	3/26	3/5
4/1	Rural youth marriage	4/43	4/71	4/8	4/24	4/48
6/23	The stability of daily rural life	4/1	3/36	4/58	4/25	3/95
3/25	Migration	3/3	2/43	2/56	2/25	2/36
5/69	Unity and solidarity of villagers	4/36	4/49	4/52	4/52	4/38
5/25	Social dignity	4/5	4/12	3/95	4/12	3/75
6/49	Access to information	4/55	4/63	4/27	4/4	3/89
6/13	Dependence on others	4/41	4/52	4/63	4/17	4/8
6/45	Cooperation and social participation	4/24	4/82	4/63	4/63	4/17
4/25	Sympathy	4/52	4/52	4/27	4/31	4/8
**Total Vulnerability**		4/17	4/2	4/22	4/1	3/92

P_i_: The amount of each parameter on a scale of 1 (least vulnerability) to 5 (most vulnerability) from the farmers' perspective.

W_j_: The relative importance of each parameter on a scale of 0 (the lowest weight) to 10 (the highest weight).

### Psychological vulnerability

The values of psychological vulnerability parameters are presented in Table 5. The following equation was used to calculate the weight of psychological parameters from the experts' point of view.


$$\begin{array}{l} \sum Wi=C_{1}\\ \sum Wi=(Wmax\times n)/2=(10\times 15)/2=75 \end{array}$$

According to the opinion of experts, the parameters of self-efficacy (6.39), social vitality (6.26) and hope (6.02) have the most importance in the vulnerability of farmers during COVID-19. In addition, the results showed that Shabester and Sarab counties have the highest level of psychological vulnerability with vulnerability values of 4.26 and 4.20, respectively (Table 4).

**Table 4:** The values and weights of farmers' psychological vulnerability parameters.

**Weight (Wj)**	**Psychological sub-parameters**	**Value**				
		**(Pj)**				
		**Varzeghan**	**Maragheh**	**Shabestar**	**Sarab**	**Tabriz**
		**Pi**	**Pi**	**Pi**	**Pi**	**Pi**
4/11	Self-esteem	3/45	3/52	4/49	3/59	3/52
6/39	Efficacy	4/41	4/9	4/27	4/41	4/37
4/52	Worry and anxiety	4/25	4/34	4/49	4/71	4/8
6/2	Hope	4/4	4/74	4/45	4/37	4/62
4/42	Risk taking	4/5	3/85	3/67	4/1	3/55
3/58	Correct and decisive decision	3/55	3/24	3/5	3/4	2/89
6/26	Social vitality	4/55	4/25	4/73	4/67	4/52
**Total vulnerability**		4/15	4/11	4/26	4/2	4/6

P_i_: The amount of each parameter on a scale of 1 (least vulnerability) to 5 (most vulnerability) from the farmers' perspective.

W_j_: The relative importance of each parameter on a scale of 0 (the lowest weight) to 10 (the highest weight).

### Environmental vulnerability

Another factor that affects farmers' vulnerability during COVID-19 is environmental factors, the total weight of Environmental parameters is calculated from the following formula.


$$\begin{array}{lll} \sum \text{Wi} & = & {\text{C}}_{1}\\ \sum \text{Wi} & = & \left(\text{Wmax}\times \text{n}\right)/2=\left(10\times 7\right)/2=\;35 \end{array}$$

According to the opinion of the studied experts, irrigation parameters of agricultural lands (5.55), proper agricultural operations (5.53) and agricultural waste (5.45) have the greatest impact on the vulnerability of farmers during COVID-19. Moreover, the general results of the vulnerability situation show that Shabestar county has the highest level of vulnerability in this dimension compared to other counties (Table 5).

**Table 5:** The values and weights of farmers' environmental vulnerability parameters.

**Weight (Wj)**	**Environmental sub-parameters**	**Value**				
		**(Pj)**				
		**Varzeghan**	**Maragheh**	**Shabestar**	**Sarab**	**Tabriz**
		**Pi**	**Pi**	**Pi**	**Pi**	**Pi**
5/45	Agricultural production waste	3/12	3/54	3/51	2/89	3/2
5/14	cropping pattern	3/41	3/19	3/72	3/45	3/12
4/15	Type of cultivation (autumn and spring)	3/73	3/98	4/22	4/8	4/1
4/52	Equipping and renovating agricultural land	3/83	3/88	3/45	3/69	3/25
5/53	Good Agricultural Practices (GAP)	2/55	2/35	2/14	2/2	2/25
4/66	Water channel drainage	3/45	3/47	3/66	3/82	3/92
5/55	Irrigation of agricultural lands	3/71	3/25	3/47	3/66	3/51
**Total Vulnerability**		3/37	3/34	3/43	3/32	3/25

P_i_: The amount of each parameter on a scale of 1 (least vulnerability) to 5 (most vulnerability) from the farmers' perspective.

W_j_: The relative importance of each parameter on a scale of 0 (the lowest weight) to 10 (the highest weight).

### Total vulnerability

Table 6 shows the values of total vulnerability in the studied counties. According to the presented results, Shabestar and Maragheh counties have the highest and lowest levels of vulnerability, respectively. As well as, compared to Tabriz county, which was considered as a reference county, similar results were obtained with the state of vulnerability.

**Table 6:** Total vulnerability coefficients of the studied counties.

**Total Vulnerability parameters (Vi)**	**Value**			
	**(Pj)**			
	**Varzeghan**	**Maragheh**	**Shabestar**	**Sarab**	**Tabriz**
	**Pi**	**Pi**	**Pi**	**Pi**	**Pi**
Economical	3/34	3/5	3/48	3/49	3/26
Social	4/17	4/2	4/22	4/1	3/92
Psychological	4/15	4/11	4/26	4/2	4/6
Environmental	3/37	3/34	3/43	3/32	3/25
Total vulnerability (V_L_)	3/79	3/85	3/89	3/83	3/65
Relative vulnerability (V_RL_)	1/3	1/5	1/6	1/4	^a^1

a: Tabriz was considered the reference.

## Discussion

This study was conducted for vulnerability assessment of rural-farmer households in East Azerbaijan Province (northwest of Iran). Vulnerability assessment leads policy makers to know about the threats so that they can implement proper control and management. Moreover, one of the other advantages of identifying causes of vulnerability is targeted support to increase their resilience in rural communities. Therefore, the main objective of this study was to question the policies of the same support for rural communities because rural communities with different conditions usually require unique policies and environmental conditions. To that end, the present study was conducted aimed to identify the causes of vulnerability of rural households in economic, environmental, social and psychological dimensions and can help rural development planners. In the following, the most important causes of vulnerability are presented according to the study results.

The results showed that for economic vulnerability, the rural poverty was the most important cause of vulnerability of the studied rural households. This finding was consistent with studies (72). For the analysis of this finding, it can be said that poverty has plagued the world for a long time and has been one of the main concerns of the development of human societies (73). COVID-19 pandemic in 2020 has had a very great effect on the global socio-economic development, and achieving objectives of the United Nations Sustainable Development in 2030, which is the eradication of poverty, has been difficult for many countries, especially third world countries (72). During COVID-19 pandemic, poverty has become much more than before the disease, for example, a study in China showed that during the disease, poverty among households increased by 27.9% (72). The increase in poverty at the time of disease will lead to higher vulnerability of rural households in dealing with this disease because rural households will not be able to buy a variety of foods, vegetables and fruits suitable for increasing their immune system in dealing with this shock (49). Moreover, the results showed that agricultural income was the second cause of economic vulnerability of farmer households. Studies (63, 74) support this finding. For the analysis of this finding, it can be said that during COVID-19 pandemic, agricultural income of rural households reduced significantly due to severe quarantines. Moreover, many members of rural households who work in sectors related to employment in agriculture (future and previous activities) stop working due to COVID-19 pandemic and lost most of their income. Most households in these sectors, especially informal workers, do not have a secure support for hard times and hence, the loss of income will affect their food security and nutrition (43) As a study on the effect of COVID-19 pandemic in the first quarter of 2020 showed that 3.11% of the total volume of agricultural production in Southeast China has reduced (74). Studies conducted in Uganda have shown that the welfare of rural households has reduced due to the quarantine of COVID-19 pandemic (75). Because the income of farmers has reduced due to COVID-19 pandemic (63). Other studies have shown that many farmers faced many problems in marketing their products (76) and due to lack of purchase in consumer markets, the prices of many farmers' products have reduced (77). Therefore, it can be said that the quarantines caused by COVID-19 pandemic have caused the biggest disruption in livelihood (78). Furthemore, the results showed that the third cause of economic vulnerability of rural-farmer households studied in Iran was the savings of rural households. Studies (35) support this finding. For the analysis of this finding, it can be said that rural households lost few savings they had due to the constant quarantine due to their weak economic support and were forced to sell many of their capital goods such as agricultural land and livestock. Sale of the assets of rural households will increase their level of vulnerability more than ever. Moreover, the sale of assets due to the reduction in the income of agricultural households during COVID-19 pandemic will reduce the dependence of agricultural households on the village and continue to operate in the rural sector, which will finally lead to the migration of rural households from this sector, which itself can cause more damage to rural households.

The results of social vulnerability showed that access to information, social cooperation and participation and the stability of daily rural life have the greatest effect on the vulnerability of the studied wheat farmers. This finding was consistent with studies (46, 47, 49). For the analysis of the results of this section, it can be said that rural households were severely affected by this disease due to the lack of access to proper information and how to deal with the shock of COVID-19 pandemic (45). In fact, rural areas had more complicated conditions compared to urban areas due to less readiness (47). As well as, due to social distancing and restrictions, participation and cooperation of rural communities has become much less, but since agricultural activity in rural communities depends on the principles of social participation and solidarity, many agricultural and social activities are closed and increased the vulnerability of rural households (79). Furthermore, due to the low access to medical services in rural communities, the rate of death in rural areas was higher than in some urban areas. Moreover, due to the disruption in the production system and livelihood sources of farmers and the destruction of the food supply chain, the stability of life reduced in rural communities (44, 55).

Furthermore, the results of environmental vulnerability showed that the most important causes of vulnerability in this sector included irrigation, proper agricultural operations, and crop waste production. This finding was consistent with the studies (17, 63). In the analysis of this finding, it can be said that during COVID-19 pandemic, due to severe quarantine, farmers could not easily carry out their agricultural activities such as irrigation, because many of these activities required collective and shared activities. Irrigation of agricultural lands is one of the most important principles of improving the performance of the agricultural sector. In fact, water is the most important input of agricultural activities (20). But this activity was disrupted at the time of this disease, for instance, a study in Uganda showed that the most important damage caused by COVID-19 pandemic was the reduction in irrigation of crops due to severe quarantine, and many farmers due to the anxiety caused by COVID-19 pandemic reduced the number of irrigations times (63). Reducing the irrigation and water input will lead to a reduction in yield and finally the income of farmers and an increase in their vulnerability. Moreover, the study results showed that the second cause of environmental vulnerability of farmers was the lack of proper agricultural operations during COVID-19 pandemic. For the analysis of this finding, it can be said that in order to perform suitable agricultural operations, farmers needed suitable production inputs, due to the closure of factories and the heavy increase in the cost of production inputs, many farmers were forced not to use these inputs much (62). In addition, due to the seasonality of planting, ripening and harvesting, proper agricultural production activities require suitable and quality seasonal workforce, which during COVID-19 pandemic due to the quarantine and anxiety caused by the disease, many agricultural activities were not done in the right way and at the right time, and it imposed a lot of crop waste on the agriculture (17). The third cause of environmental vulnerability in the studied region was agricultural production waste. Many agricultural activities due to many problems such as reduced exports, lack of workforce, harvest time, and other production activities had production waste because suitable agricultural operations were not performed (58). Moreover, in some rural areas, they do not have the proper infrastructure for transporting the product and the product is faced with a lot of waste (14, 80). Therefore, in general, it can be said that crop waste leads to the loss of part of the income of rural households and their level of resilience in dealing with disease reduces.

Finally, the results showed that the three parameters of self-efficacy, social vitality and hope are the most important parameters for the psychological vulnerability of farmers. Studies (81, 82) support this finding. For the analysis of the results of this section, it can be said that the studied farmers usually do not have sufficient self-efficacy due to their lower education level (20). According to definition of self-efficacy, which refers to the extent to which a person feels that he can protect and deal with COVID-19 pandemic, in other words, refers to the ability to use preventive and healthy behaviors to deal with COVID-19 pandemic (29). Therefore, if farmers consider preventive behaviors as simple and possible, it is possible to use coping behaviors (81). On the other hand, applying restrictions such as quarantine during the disease increases the possibility of mental problems, and if interpersonal communication is not established, depression and anxiety are likely to occur and worsen over time, and so social vitality becomes less (11). In addition, in households who lost one of the family members due to the disease, it has a very strong psychological effect on the households and reduces the hope of improving the situation (59, 60). Moreover, other things during the disease, such as fear of infection, despair, boredom, insufficient sources, insufficient information, and severe financial loss have other psychological effects on the society and cause despair (73).

## Conclusion

Vulnerability assessment is an efficient tool for economic, social, psychological, and environmental improvement in order to achieve sustainable development. Rural areas have always been emphasized and paid attention to by policymakers due to the fact that they contain a large part of the population. The living conditions of rural households are considered important with regard to ensuring the food security of a country, as well as the pristine environment that the village provides to the public from its perspectives, as well as social ties (83). Therefore, any change in these living conditions of villagers can have many consequences on other regions of the country. The COVID-19 pandemic has left various effects on the situation of the world, especially in rural areas. Considering the pandemic of this disease in Iran, especially in East Azarbaijan province, and especially in the rural areas of this province, and considering the importance of the topic, this research has been conducted with the aim of Vulnerability Assessment of Iran's Rural-Farmer Households during COVID-19 Pandemic. In this study, using (68) model, the vulnerability of farmers was investigated in four economic, social, psychological and environmental dimensions. The model was used to investigate the level of vulnerability during COVID-19 pandemic for the first time and it can help to fill the gap of previous studies. In general, the study results showed that the studied farmers experienced high levels of vulnerability and needed targeted interventions to increase their resilience. Based on research results; factors of Rural poverty, agricultural income, and household savings are the largest source of vulnerability for wheat farmers in northwestern Iran. In addition, a glance at the economic vulnerability of wheat farmers reveals that all studied areas are at a high level of economic vulnerability as the amount of vulnerability is above the average (3 of 5) in all areas. Therefore, due to the fact that many wheat farmers suffered due to the corona disease, it is suggested that farmers can use alternative sources of livelihood when one of their sources of income is lost by improving livelihood diversity.

In another part of this research, the results showed that the parameter of access to information have the greatest effect on the social vulnerability of the studied wheat farmers. Therefore, it is suggested to provide the necessary arrangements for villagers and farmers to access information through the creation and strengthening of infrastructure related to the Internet in order to facilitate access to information.

To that end, according to the study results, three general policies are suggested to reduce the vulnerability of farmers.

1. Targeted subsidy support: It is suggested that the government provides targeted subsidy support by identifying poor and needy households so that rural households do not sell their assets and main sources of income.

2. Development of educational and medical services: Many rural households do not have sufficient knowledge of the methods of coping and social distancing and have not received sufficient training. Therefore, it is suggested to hold workshops and courses.

3. Development of agricultural operations: Many farmers do not have access to production facilities during COVID-19 pandemic. Therefore, it is suggested to provide them with suitable facilities to help maintain production in the agricultural sector.

It should be noted that due to the fact that this research was conducted during the Corona period, we had to receive information from the studied samples through an electronic questionnaire. Because of this, the researchers could not attend the field and see the condition of the farmers and villagers under study due to traffic restrictions in the Corona situation.

## Data availability statement

The raw data supporting the conclusions of this article will be made available by the authors, without unduereservation.

## Author contributions

All authors listed have made a substantial, direct, and intellectual contribution to the work and approved it for publication.

## Conflict of interest

The authors declare that the research was conducted in the absence of any commercial or financial relationships that could be construed as a potential conflict of interest.

## Publisher's note

All claims expressed in this article are solely those of the authors and do not necessarily represent those of their affiliated organizations, or those of the publisher, the editors and the reviewers. Any product that may be evaluated in this article, or claim that may be made by its manufacturer, is not guaranteed or endorsed by the publisher.
